# Human iPSCs derived from cryopreserved testicular somatic cells enable germline regeneration in childhood cancer survivors

**DOI:** 10.1093/hropen/hoag054

**Published:** 2026-06-03

**Authors:** Tiago Macedo, Claudia De Guidi, Leah Nic Aodha, Nageswara Rao Boggavarapu, Maja Piechocka, Xuan Ye, Francesca Mastropasqua, Victoria Keros, Ulrika Norén Nyström, Per Frisk, Pia Johansson, Kristiina Tammimies, Yoni Baert, Kirsi Jahnukainen, Jan-Bernd Stukenborg, João Pedro Alves-Lopes

**Affiliations:** Department of Women’s and Children’s Health, Karolinska Institutet and Karolinska University Hospital, Stockholm, Sweden; Cell and Gene Technologies Core, Lund Stem Cell Center, Lund University, Lund, Sweden; Department of Women’s and Children’s Health, Karolinska Institutet and Karolinska University Hospital, Stockholm, Sweden; Division of Obstetrics and Gynecology, Department of Women’s and Children’s Health, Karolinska Institutet, and Karolinska University Hospital, Stockholm, Sweden; Department of Women’s and Children’s Health, Karolinska Institutet and Karolinska University Hospital, Stockholm, Sweden; Department of Women’s and Children’s Health, Center of Neurodevelopmental Disorders (KIND), Centre for Psychiatry Research, Karolinska Institutet, Stockholm, Sweden; Department of Highly Specialized Pediatric Orthopedics and Medicine, Astrid Lindgren Children’s Hospital, Karolinska University Hospital, Stockholm, Sweden; Department of Women’s and Children’s Health, Center of Neurodevelopmental Disorders (KIND), Centre for Psychiatry Research, Karolinska Institutet, Stockholm, Sweden; Department of Highly Specialized Pediatric Orthopedics and Medicine, Astrid Lindgren Children’s Hospital, Karolinska University Hospital, Stockholm, Sweden; Division of Urology, Karolinska Institutet and Reproductive Medicine, Department of Clinical Science, Intervention and Technology (CLINTEC), Karolinska University Hospital, Stockholm, Sweden; Department of Clinical Sciences, Pediatrics, Umeå University, Umeå, Sweden; Department of Women’s and Children’s Health, Uppsala University, Uppsala, Sweden; Cell and Gene Technologies Core, Lund Stem Cell Center, Lund University, Lund, Sweden; Department of Women’s and Children’s Health, Center of Neurodevelopmental Disorders (KIND), Centre for Psychiatry Research, Karolinska Institutet, Stockholm, Sweden; Department of Highly Specialized Pediatric Orthopedics and Medicine, Astrid Lindgren Children’s Hospital, Karolinska University Hospital, Stockholm, Sweden; In Vitro Toxicology and Dermato-cosmetology (IVTD), Vrije Universiteit Brussel, Brussels, Belgium; Department of Women’s and Children’s Health, Karolinska Institutet and Karolinska University Hospital, Stockholm, Sweden; New Children’s Hospital, University of Helsinki and Helsinki University Hospital, Helsinki, Finland; NORDFERTIL Research Lab Uppsala, Department of Organismal Biology, Uppsala University, Uppsala, Sweden; Department of Women’s and Children’s Health, Karolinska Institutet and Karolinska University Hospital, Stockholm, Sweden; NORDFERTIL Research Lab Uppsala, Department of Organismal Biology, Uppsala University, Uppsala, Sweden; Department of Women’s and Children’s Health, Karolinska Institutet and Karolinska University Hospital, Stockholm, Sweden

**Keywords:** testicular tissue cryopreservation, somatic cell reprogramming, induced pluripotent stem cells, primordial germ cells, paediatric oncology, gonadotoxicity, infertility, fertility preservation, *in vitro* gametogenesis (IVG), regenerative medicine

## Abstract

**STUDY QUESTION:**

Can cryopreserved primary testicular somatic cells from childhood cancer survivors with severely decreased fertility potential be reprogrammed into human-induced pluripotent stem cells (hiPSCs) competent for efficient specification into early human germ cells?

**SUMMARY ANSWER:**

Primary testicular somatic cells from cryopreserved testicular samples with severely compromised spermatogonial pools can be reprogrammed into hiPSCs using a non-genome-integrating, feeder-free approach and subsequently differentiated into human primordial germ cell-like cells (hPGCLCs) with high efficiency.

**WHAT IS KNOWN ALREADY:**

Infertility is one of the most concerning long-term side effects of cancer therapy in prepubertal boys, yet it remains largely unaddressed. Worldwide, biobanks storing cryopreserved immature testicular tissue are expanding to support the development of fertility preservation strategies, including future tissue and cell transplantation approaches. However, whether these samples can also serve as starting material to generate hiPSCs and subsequently differentiate into *in vitro*-derived germ cells remains largely unexplored. Currently, there are established protocols for non-genome-integrating reprogramming of somatic cells into hiPSCs and for hPGCLC specification that could be applied to cryopreserved testicular tissue, with special relevance for patient samples severely depleted of germ cells.

**STUDY DESIGN, SIZE, DURATION:**

Two biological replicates of cryopreserved prepubertal testicular tissue were used to obtain primary somatic cells, which were reprogrammed into hiPSCs and subsequently differentiated into hPGCLCs. This experimental pipeline had a duration of approximately four months.

**PARTICIPANTS/MATERIALS, SETTING, METHODS:**

Cryopreserved testicular tissue samples were obtained from two prepubertal cancer patients (6.2 and 6.3 years old) with depleted spermatogonial pools (spermatogonia count per round tubular cross-section of 0.04 and 0.02, and age-standardized *Z*-scores of −17.62 and −20.48). These samples were subsequently used to derive primary testicular somatic cell cultures, which were then reprogrammed into hiPSCs using a clinically compatible, non-genome-integrating mRNA-based method under feeder-free conditions. The resulting hiPSC lines were validated and subsequently differentiated into hPGCLCs using two different specification protocols. The transcriptomic profiles of the hiPSCs and their derived hPGCLCs were verified using bulk RNA sequencing.

**MAIN RESULTS AND THE ROLE OF CHANCE:**

Here, we present the first successful generation of hiPSCs from cryopreserved testicular somatic cells of childhood cancer patients with a severely depleted germ cell pool. We accomplished this using a non-genome-integrating mRNA-based reprogramming approach. We further demonstrated the specification of hPGCLCs from these patient-derived hiPSCs, effectively regenerating their germline. This provides proof-of-concept for a stem cell-based fertility regeneration strategy in childhood cancer survivors with non-functional or absent germ cells.

**LARGE SCALE DATA:**

N/A

**LIMITATIONS, REASONS FOR CAUTION:**

This proof-of-concept study was limited by a small sample size due to the restricted access to cryopreserved human prepubertal testicular tissues. Although multiple hiPSC clones were generated per individual, only one clone per patient was used for downstream analyses, precluding systematic assessment of intra-individual clonal variability, which remains an important aspect for future studies. While G-banding karyotyping was sufficient to validate genomic integrity in this proof‑of‑concept study, more comprehensive genetic and epigenetic analyses should be prioritized in future hiPSC and hPGCLC validation before any clinical application is considered.

**WIDER IMPLICATIONS OF THE FINDINGS:**

Childhood cancer patient-derived hiPSCs represent a powerful platform to investigate and address a broad range of long-term, cancer therapy-related complications. These hiPSCs and their derived germ cells may be used not only to develop *in vitro* gametogenesis protocols, but also to investigate mechanisms of cancer therapy toxicity and resistance among different patients, to identify early biomarkers of adverse outcomes, and to screen for germ cell protective agents. Importantly, cancer patient-specific hiPSCs have applications that extend well beyond germline, enabling regenerative strategies targeting other treatment-related sequelae through the generation of relevant somatic cell types.

**FUNDING:**

T.M. was supported by the Erasmus+ program, as part of the projects WORK4ALL 2023 and WORK4ALL 2024 (2023-1-PT01-KA131-HED-000121324 and 2024-1-PT01-KA131-HED-000214636). L.N.A. was supported by a Marie Skłodowska-Curie Actions Individual Fellowship (101278886: GERMFIT) from the European Commission. Y.B. was supported by the Scientific Fund Willy Gepts. K.J. was supported by the Foundation for Pediatric Research, the Finnish Cancer Society, the Swedish Childhood Cancer Foundation (KP2020-0012), and the Birgitta and Carl-Axel Rydbeck’s Research Grant for Paediatric Research (2020-00335, 2021-00079, and 2023-00380). J.-B.S. was supported by the Swedish Childhood Cancer Fund (PR2019-0123; PR2022-0115; TJ2020-0023) and the Swedish Research Council (2018-03094; 2021-02107). J.P.A.-L. was supported by a Starting Grant in Medicine and Health (2022-01467) from the Swedish Research Council, the Birgitta and Carl-Axel Rydbeck Research Grant for Paediatric Research 2024 (2024-00208), and the Scientific Fund Willy Gepts.

**DISCLOSURES:**

All authors declare no conflicts of interest.

WHAT DOES THIS MEAN FOR PATIENTS? Prepubertal boys who undergo cancer treatment face a high risk of lifelong infertility because the cells that normally produce sperm can be damaged or lost. In this study, we explored whether other testicular cells could be used to help restore these damaged germ cells. Using preserved samples from two young cancer patients, we reprogrammed testicular supporting cells into stem cells, special cells capable of developing into any cell type. We then guided these stem cells to develop into early-stage germ cells, the precursors of sperm. Our results demonstrate, for the first time, that even when sperm-producing cells are severely reduced, it is still possible to generate early germ cells from stored testicular tissue. In the short term, this work will help us better understand how cancer treatments damage reproductive cells and how to protect them. In the long term, it paves the way for new regenerative approaches to fully restore fertility in cancer survivors, expanding current assisted reproduction options.

## Introduction

Childhood cancer remains a leading cause of mortality among children and adolescents in Europe, with approximately 10 000 new diagnosed cases and over 1000 deaths each year (Bertuccio *et al.*, 2020; ECIS—European Cancer Information System, 2024). While advances in diagnostics and treatment regimens have dramatically improved survival rates over the past decades, the long-term side effects of cancer therapies remain insufficiently addressed (Stenmarker *et al.*, 2024). These impose a lasting burden on the quality of life of survivors, with over 80% estimated to develop a serious chronic condition by the age of 45 (Hudson *et al.*, 2013; Bhakta *et al.*, 2017; Koskela *et al.*, 2025).

In male survivors, infertility is among the most impactful and under-discussed sequelae, profoundly affecting their future family planning and psychosocial well-being (Dionisi-Vici *et al.*, 2023; El Alaoui-Lasmaili *et al.*, 2023). Long-term follow-up studies during adulthood reveal an increased prevalence of fertility-related problems, with the degree of damage varying by treatment type, cumulative dose, and patient age at exposure (Wasilewski-Masker *et al.*, 2014; Holmer *et al.*, 2024). Although predicting individual fertility outcomes based on planned therapy regimens remains challenging, treatment with alkylating agents and whole-body irradiation are known to pose the highest threat of gonadotoxicity (Green *et al.*, 2010; Poorvu *et al.*, 2019; Funke *et al.*, 2021), representing the main criteria for the eligibility of fertility preservation techniques in prepubertal boys (Mulder *et al.*, 2021; Mitchell *et al.*, 2025).

Unlike postpubertal males, who can benefit from established sperm cryopreservation techniques (Hughes and Martins da Silva, 2022), the lack of mature sperm in prepubertal boys restricts fertility preservation efforts to experimental approaches (Vogt and Malhotra, 2025). Over the past two decades, an increasing number of medical centres worldwide have begun cryopreserving immature testicular tissue from prepubertal patients undergoing gonadotoxic therapies, aiming to develop future fertility treatment strategies (Duffin *et al.*, 2024). Early estimates suggest that approximately a quarter of these patients will later depend on their cryopreserved samples to achieve biological parenthood (Wyns *et al.*, 2021). Yet, although testicular tissue cryopreservation represents a major option in male reproductive health, the spermatogonial pool may already be severely compromised prior to the procedure (Stukenborg *et al.*, 2018). For clinical and logistical reasons, timely access to prepubertal testicular tissue cryopreservation cannot be ensured for all paediatric cancer patients. In addition, reduced spermatogonial numbers have been reported in boys with severe haematological conditions even before gonadotoxic therapy (Lahtinen *et al.*, 2024). Moreover, many survivors at high risk of future infertility remain unaware of, or encounter barriers accessing fertility preservation programs, highlighting a critical gap in long-term survivorship care (Brungardt *et al.*, 2022).

Potential applications making use of the preserved testicular tissue to restore fertility rely on the presence of viable spermatogonial stem cells (SSCs), the pivotal germ cell pool capable of self-renewal and sequential differentiation into mature spermatozoa (Diao *et al.*, 2022). While earlier attempts to autologously transplant cryopreserved testicular tissue or SSCs failed to demonstrate germ cell survival or spermatogenesis (Jensen *et al.*, 2024; Zielen *et al.*, 2025), a recent proof-of-concept study showed that cryopreserved prepubertal human testicular tissue can survive long-term storage, revascularize after autologous transplantation, and support spermatogenesis (Goossens *et al.*, 2026). Further challenges stem from the absence or low viability of SSCs in the tissue, particularly when samples are collected after the initiation of chemotherapy or irradiation (Valli-Pulaski *et al.*, 2019; Funke *et al.*, 2021). Given the strong preference for biological parenthood among infertile couples (Hendriks *et al.*, 2017), this limitation raises pressing concerns about the lack of viable fertility restoration options for patients facing the most severe testicular germ cell sequelae.

In this context, generating SSCs from patient-specific induced pluripotent stem cells (iPSCs) offers the most compelling alternative. Since the inception of hiPSC generation from the viral delivery of key pluripotency factors to dermal fibroblasts (Takahashi *et al.*, 2007), novel techniques have addressed practical, safety, and efficacy concerns, allowing broad accessibility of hiPSC-based therapies (Cerneckis *et al.*, 2024; Kirkeby *et al.*, 2025; Macedo and Alves-Lopes, 2026). These include mRNA vectors, allowing non-genome-integrating reprogramming of somatic cells, as well as novel highly scalable small molecule-based protocols (Cheng *et al.*, 2025).

Over the past decade, there have been significant advances in reconstituting the human male germline from pluripotent stem cells (PSCs), particularly with the ability to generate human primordial germ cell-like cells (hPGCLCs), the *in vitro* equivalent of human primordial germ cells (hPGCs), which represent the earliest, most immature germ cells occurring in humans (Saitou *et al.*, 2026). These protocols, developed to overcome the extremely limited access to human embryonic material at this developmental stage, were vital to uncovering some of the mechanisms involved in early germline development (Ramakrishna *et al.*, 2025). Traditionally, hPSCs are first induced into either peri-gastrulation precursors (representing epiblast cells at the onset of gastrulation, approximately during developmental days (E) 13–16), or primed/naïve intermediary state precursors (representing epiblast cells between the naïve and primed states of pluripotency, ∼E9–12) before being induced into the hPGC fate (Macedo and Alves-Lopes, 2026). Subsequent maturation efforts have advanced hPGCLCs to prospermatogonia, bringing us closer to recapitulating late embryonic stages of male gametogenesis *in vitro* (Hwang *et al.*, 2020; Murase *et al.*, 2024). As the field approaches the milestone of further maturing *in vitro*-derived germ cells to the state of SSC (Whelan *et al.*, 2024), there is growing attention to the ethical, social, and clinical safety issues that accompany this emerging reproductive technology (de Bruin *et al.*, 2025; Macedo and Alves-Lopes, 2026).

**Figure 1.**
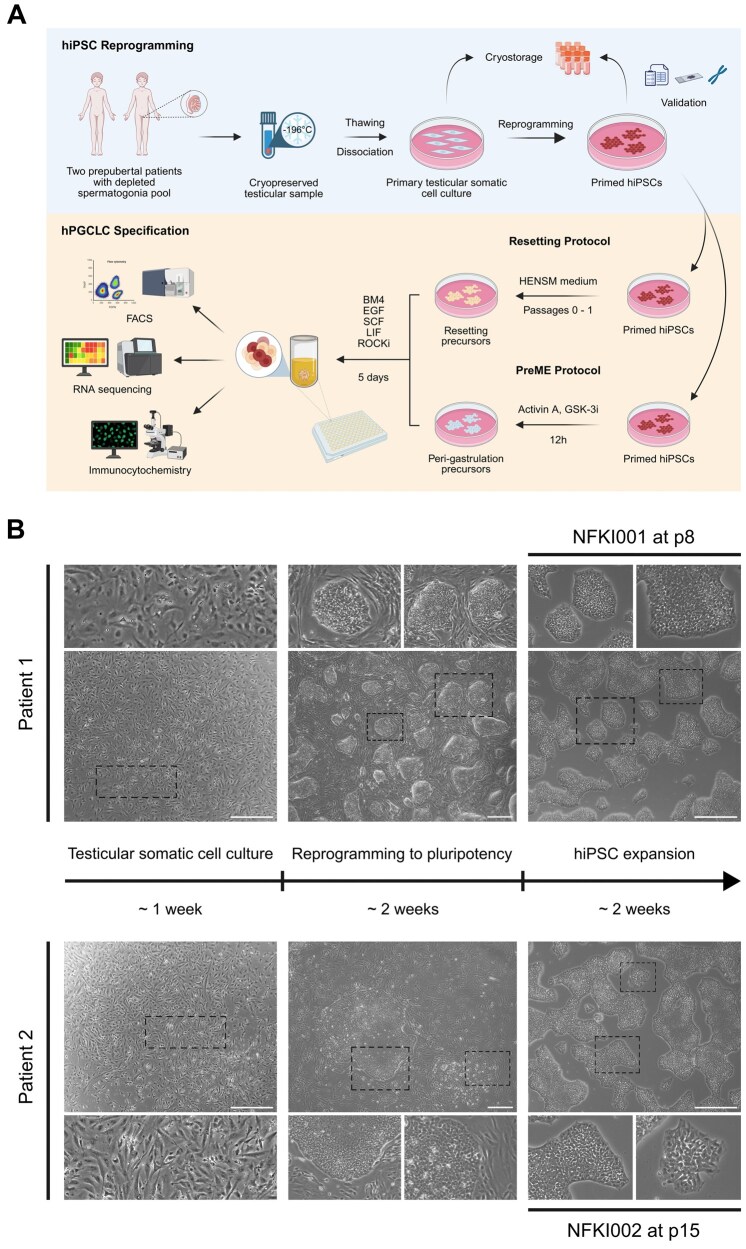
**Overview of the experimental layout: focus on primary testicular somatic cell cultures and reprogramming**. (**A**) Schematic overview of the experimental procedures. (**B**) Non-genome-integrating, feeder-free reprogramming of human induced pluripotent stem cells (hiPSCs) from primary testicular somatic cells, with the corresponding time required for each step. Left panel: Phase-contrast images of confluent primary testicular somatic cell cultures approximately 1 week after isolation from the cryopreserved tissue; Middle panel: Emerging hiPSC colonies following the mRNA-based reprogramming protocol; Right panel: Expansion of the isolated hiPSC clones NFKI001 at passage 8 (p8) and NFKI002 at p15 from Patients 1 and 2, respectively. Scale bars, 500 μm. Figure (A) was created using BioRender (Alves Lopes, J. P. (2026); https://BioRender.com/gv6k171).

In this study, we report for the first time the successful generation of hiPSCs from cryopreserved testicular tissue of childhood cancer patients with a severely compromised spermatogonial pool, using a non-genome-integrating mRNA-based reprogramming strategy. Furthermore, we show that these patient-specific hiPSCs can be differentiated into hPGCLCs, effectively regenerating their germline and providing a proof of concept for a fertility-restoration approach in childhood cancer survivors who lack viable SSCs (Fig. 1A).

## Materials and methods

### Ethics statement

The use of testicular tissue to develop *in vitro*-based strategies for fertility preservation, including hiPSC reprogramming for germline specification, described in this article, is covered under the ethical approvals from the Regional Ethics Board in Stockholm (Dnr 2013-2129-31-3 and Dnr 2021-04277).

### Testicular tissue processing and primary testicular tissue cell expansion

Testicular samples were obtained from two prepubertal patients who participated in the Nordic Centre for Fertility Preservation (NORDFERTIL) fertility preservation project. Participants were identified as having a high risk of treatment-induced infertility due to planned therapeutic interventions, including allogeneic or autologous hematopoietic cell transplantation or radiotherapy involving the testes. Exclusion criteria included evidence of ongoing spermatogenesis (testicular volumes >10 ml, assessed by orchidometry) and a significant risk of bleeding or infection. At the time of sample retrieval, Patient 1 was 6.3 years old and had received a cumulative cyclophosphamide equivalent dose (CED) of 4814 mg/m^2^ for the treatment of a solid tumour. Patient 2 was 6.2 years old and received a cumulative CED dose of 16 000 mg/m^2^ for the treatment of a solid tumour. Reflecting the gonadotoxic impact of their treatment, Patients 1 and 2 exhibited a severely depleted spermatogonial pool, with a mean spermatogonia count per round tubular cross-section (S/T) of 0.04 and 0.02, and age-standardized *Z*-scores of −17.62 and −20.48, respectively, both substantially below the threshold for severe depletion. According to published reference values, S/T *Z*-scores between −3 and +3 SD are considered within the normal range for spermatogonial numbers, between −3 and −7 SD considered reduced level, whereas scores below −7 SD indicate pronounced spermatogonial depletion (Funke *et al.*, 2021).

Testicular tissue samples were retrieved by open biopsy and, after equilibration in a cryoprotective medium containing 0.7 M DMSO, were cryopreserved using a conventional slow programmed freezing protocol (Keros *et al.*, 2007). According to established experimental procedures, two-thirds of each testicular biopsy were cryopreserved for fertility preservation, while one-third was donated for research and either processed fresh or similarly cryopreserved in multiple vials. The present study utilized cryopreserved samples from the fraction allocated for research. After thawing the cryopreserved prepubertal testicular tissue fragments (Keros *et al.*, 2005), roughly one-tenth to one-twentieth of the sample was minced with a scalpel and digested enzymatically with a 30-minute incubation at 37°C in 0.25% Trypsin–EDTA (Thermo Fisher Scientific, Waltham, MA, USA, #25200056). The reaction was neutralized with an equal volume of stopping medium, consisting of DMEM (Thermo Fisher Scientific, #10569010), 10% v/v foetal bovine serum (Sigma-Aldrich, St. Louis, MO, USA, # F7524), and 100 U/ml Penicillin–Streptomycin (Thermo Fisher Scientific, # 15140122).

The resulting cell suspension was centrifuged at 300 *g* for 3 min and resuspended in a fibroblast-supporting medium [DMEM, high glucose, GlutaMAX™ Supplement, pyruvate, 10% v/v foetal bovine serum, 1× MEM non-essential amino acids (Thermo Fisher Scientific, #11140050) and 100 U/ml Penicillin–Streptomycin] supplemented with 10 μM of Y-27632 dihydrochloride (Tocris Bioscience, Bristol, UK, #1254). Given the mixed cellular composition of the initial digest, with Sertoli and interstitial cells being the predominant cell types (unpublished data), the resulting cultures are referred to as primary testicular somatic cells, consistent with previous reports (Martin-Inaraja *et al.*, 2025; You *et al.*, 2025). These were seeded in 24-well plates (Sarstedt, Nümbrecht, Germany, #83.3922) pre-coated with either 300 μl of Attachment Factor 1× (Thermo Fisher Scientific, # S006100) or 20 μg/cm^2^ of Matrigel, GFR, LDEV-free (Corning, NY, USA, #354230) and maintained at 21% O_2_, 5% CO_2_ at 37°C. The medium was replaced every second day. Cells were passaged with 0.25% Trypsin–EDTA at split ratios of 1:4 to 1:2 upon ∼85% confluence. Cultures were expanded for 2–4 passages in 12-well plates before cryopreservation in CELLBANKER1 medium (Zenogen Pharma, Fukushima, Japan, #11910). All phase-contrast images of primary testicular somatic cell cultures were captured using an Axio Vert.A1 inverted microscope (ZEISS, Oberkochen, Germany) and a monochromatic Axiocam 506 mono camera (ZEISS).

### hiPSC generation and maintenance

Prior to the reprogramming, primary testicular somatic cells were thawed and cultured in fibroblast-supporting medium on rhLaminin-521 (Thermo Fisher Scientific, #A29248) coated plates. Upon reaching confluency, cells were seeded at 2000 cells/cm^2^ in 35 mm dishes on day-3 and transfected twice daily from day 0 to day 4 using the StemMACS™ iPSC mRNA Reprogramming Kit (Miltenyi Biotec, Bergisch Gladbach, Germany, #130-132-990). The medium was replaced daily and supplemented until day 14 with 3 μM of the DOT1L inhibitor EPZ-5676 (MedChemExpress, Monmouth Junction, NJ, USA, #HY-15593) and 1 μM of the CREBBP/EP300 inhibitor SGC-CBP30 (MedChemExpress, #HY-15826) to enhance reprogramming efficacy (Onder *et al.*, 2012; Ebrahimi *et al.*, 2019; Wille and Sridharan, 2022). Between days 8 and 11, cells began adopting a rounded epithelial morphology and clustering together, giving rise to stable hiPSC colonies with defined edges at days 12–14. Eight different colonies were manually picked for passage 1 and expanded in Essential 8 medium (E8; Thermo Fisher Scientific, #A1517001) on rhLaminin-521-coated wells at 21% O_2_, 5% CO_2_ at 37°C, with daily medium replacement. Subsequent cultures were maintained in vitronectin (Thermo Fisher Scientific, #A14700) coated wells, and cells were passaged with TrypLE™ (Thermo Fisher Scientific, #12604013) at split ratios of 1:20 to 1:10 upon ∼80% confluency. After splitting, the medium was supplemented with 10 μM Y-27632 for 24 h.

### Karyotyping

G-banding karyotype analysis was performed at the Clinical Genetics Department, Karolinska University Hospital. Normal karyotypes were confirmed in 20 out of 20 cells for both hiPSC lines generated in this study.

### Immunocytochemical staining for pluripotency markers

hiPSCs were seeded on vitronectin-coated 8-well µ-Slides (Ibidi, Gräfelfing, Germany, #80826) at a density of 10 000 cells/cm^2^ in Essential 8 medium, supplemented with 10 μM Y-27632. After 2–3 days, the culture was washed with DPBS (Thermo Fisher Scientific, #14190144) and fixed in 4% paraformaldehyde (Sigma-Aldrich, #P6148) for 15 min at room temperature. Cells were permeabilized with 0.1% v/v Triton X-100 in PBS for 2 h and blocked with 10% v/v normal donkey serum (ImmunoReagents, Raleigh, NC, USA, #SP-072-VX2) for 1 h. Samples were incubated overnight at 4°C with 1:200 goat anti-OCT3/4 (Santa Cruz Biotechnology, Dallas, TX, USA, #sc-8628; RRID: AB_653551) and 1:200 rabbit anti-SOX2 (Abcam, Cambridge, UK, #ab97959; RRID: AB_2341193). After washing, cells were incubated for 1 h at room temperature with 1:500 Donkey anti-Goat IgG Alexa Fluor™ 488 (Invitrogen, Thermo Fisher Scientific, #A11055; RRID: AB_2534102), 1:500 Donkey anti-Rabbit IgG Alexa Fluor™ 568 (Invitrogen, Thermo Fisher Scientific, #A10042; RRID: AB_2534017), and 1 µg/ml of DAPI (Sigma-Aldrich, #D9542). Cells were imaged using the LSM 900 confocal microscope (ZEISS).

### Pluripotency validation via trilineage differentiation capability

Trilineage differentiation was performed with the STEMdiff™ Trilineage Differentiation Kit (STEMCELL Technologies, Vancouver, BC, Canada, #05230) according to the manufacturer’s instructions. Briefly, 50 000 (for mesoderm differentiation) or 200 000 (for ectoderm and endoderm differentiation) hiPSCs/cm^2^ were seeded in Matrigel-coated 8-well µ-Slides. Differentiation media volumes were adjusted to 500 µl and replaced daily for 5 days (mesoderm and endoderm) or 7 days (ectoderm).

Cells were fixed and immunocytochemically stained as described above using antibodies against lineage markers: Ectoderm—1:200 rabbit anti-PAX6 (Thermo Fisher Scientific, #42-6600; RRID: AB_2533534) and 1:1000 mouse anti-Nestin (Merck Millipore, Darmstadt, Germany, #MAB5326; RRID: AB_2251134); Mesoderm—1:1500 rabbit anti-Brachyury (Cell Signaling Technology, Danvers, MA, USA, #81694; RRID: AB_2799983) and 1:500 mouse anti-NCAM1 (Cell Signaling Technology, #3576; RRID: AB_2149540); Endoderm—1:250 rabbit anti-FOXA2 (Thermo Fisher Scientific, #710730; RRID: AB_2576440) and 1:200 goat anti-SOX17 (R&D Systems, Minnneapolis, MN, USA, #AF1924; RRID: AB_355060). Following overnight incubation and washing, cells were incubated for 1 h at room temperature with combinations of Donkey anti-Goat IgG Alexa Fluor™ 488 (Invitrogen, Thermo Fisher Scientific, #A11055), Donkey anti-Mouse IgG Alexa Fluor™ 488 (Invitrogen, Thermo Fisher Scientific, #A21202; RRID: AB_141607), and Donkey anti-Rabbit IgG Alexa Fluor™ 568 (Invitrogen, Thermo Fisher Scientific, #A10042; RRID: AB_2534017) at 1:500. DAPI was used at 1 µg/ml. Imaging was performed using a LSM 900 confocal microscope.

### hPGCLC specification

The specification of hPGCLCs from the generated hiPSC lines was performed using embryoid body (EB)-based protocols, starting from 2 different precursor states (Alves-Lopes *et al.*, 2024).

To obtain peri-gastrulation precursors, we used the mesendoderm precursor (PreME) protocol (Kobayashi *et al.*, 2017). Briefly, primed hiPSCs growing in Essential 8 medium were cultured for 12 h at a density of 55 000 cells/cm^2^ in aRB27 basal medium [RPMI 1640 Medium (Thermo Fisher Scientific, #21870076), 1× B27 supplement (Thermo Fisher Scientific, #17504044), 2 mM l-Glutamine (Thermo Fisher Scientific, #25030081), 1× MEM non-essential amino acids, and 100 U/ml penicillin–streptomycin], supplemented with 100 ng/ml Activin A (Thermo Fisher Scientific, #120-14-10UG), 3 μM CHIR99021 (Tocris, #4423), and 10 μM Y-27632 under 21% O_2_, 5% CO_2_ at 37°C for mesendodermal differentiation.

To obtain resetting precursors, we used the human enhanced naïve stem cell media (HENSM) protocol, in which primed hiPSCs are prompted towards naïve pluripotency, giving rise to an intermediate precursor state amenable to hPGCLC specification (Bayerl *et al.*, 2021; Alves-Lopes *et al.*, 2023). Briefly, primed hiPSCs were transferred into HENSM medium at a density of 330 000 cells/cm^2^ under 21% O_2_, 5% CO_2_ at 37°C [1:1 mixture of Neurobasal (Thermo Fisher Scientific, #21103049) and DMEM/F-12 media (Thermo Fisher Scientific, #21331020), supplemented with 1× N-2 supplement (Thermo Fisher Scientific, #17502048), 2 mM l-Glutamine, 1× MEM non-essential amino acids, 100 U/ml penicillin–streptomycin, 1× B-27 supplement, 0.8 mM Dimethyl 2-oxoglutarate (Sigma-Aldrich, #349631), 0.2% v/v Matrigel, 50 mg/ml L-Ascorbic acid 2-phosphate sesquimagnesium salt hydrate (Sigma-Aldrich, #A8960), 20 ng/ml hLIF (Thermo Fisher Scientific, #300-05-25UG), 1 μM PD0325901 (Axon Medchem, Groningen, Netherlands, #1408), 2 μM XAV939 (Axon Medchem, #1527), 0.8 μM BIRB0796 (Axon Medchem, #1358), 2 μM Gö6983 (Axon Medchem, #2466), 1 μM Y-27632, 1.2 μM CGP77675 (Axon Medchem, #2097) and 20 ng/mL Activin A]. For the first 24 h, the medium was further supplemented with 5 μl Matrigel and 10 μM Y-27632. Following the 48 h culture at passage 0 (p0), cells were dissociated with Accutase (Thermo Fisher Scientific, #A1110501) and replated in the same conditions with a split ratio of 1:2 for p1.

After the respective culture periods, precursors were dissociated into single cells and seeded into an ultra-low attachment 96-well plate (Sarstedt, #83.3926) at a density of 4500 (peri-gastrulation precursors) or 5500 (resetting precursors) live cells per well. The resetting precursors were collected at both p0 and p1. Human PGCLCs were specified in aRB27 basal medium supplemented with 100 ng/ml BMP4 (Thermo Fisher Scientific, #AF-120-05ET, 10UG), 10 ng/ml LIF, 100 ng/ml SCF (Thermo Fisher Scientific, #300-07-50UG), 100 ng/ml EGF (R&D Systems, #236-EG), and 10 μM Y-27632 for 5 days under 21% O_2_, 5% CO_2_ at 37°C.

### Immunofluorescence staining for hPGCLC markers

The resulting EBs were fixed in 4% paraformaldehyde for 2 h at 4°C, cryoprotected by overnight incubations in 10% and 20% sucrose in PBS, embedded in OCT (Sakura Finetek, Torrance, CA, USA, #4583), and cryosectioned with a thickness of 8 μm (Thermo Scientific NX70 CryoStar). Sections were subjected to antigen retrieval in Tris–EDTA buffer (pH 8) at 95°C for 15 minutes, permeabilized with 0.1% Triton X-100, and blocked with 10% v/v normal donkey serum. Samples were incubated overnight at 4°C with 1:200 goat anti-SOX17 (R&D Systems, #AF1924; RRID: AB_355060), 1:200 rabbit anti-TFAP2C (Abcam, #ab76007; RRID: AB_1309954), and 1:200 mouse anti-OCT4 (BD Biosciences, Franklin Lakes, NJ, USA, #611203; RRID: AB_398737). Secondary antibodies included 1:500 Donkey anti-Goat IgG Alexa Fluor™ 488 (Invitrogen, Thermo Fisher Scientific, #A11055; RRID AB_2534102), 1:500 Donkey anti-Rabbit IgG Alexa Fluor™ 568 (Invitrogen, Thermo Fisher Scientific, #A10042; RRID: AB_2534017), and 1:500 Donkey anti-Mouse IgG Alexa Fluor™ 647 (Invitrogen, Thermo Fisher Scientific, #A31571; RRID: AB_162542), with DAPI counterstaining at 1 μg/ml. Slides were mounted in ProLong-Gold antifade reagent (Thermo Fisher Scientific, #P36930) and imaged using an LSM 900 confocal microscope.

### hPGCLC sorting and analysis

Day 5 EBs were washed with PBS and dissociated to single cells with sequential incubations in 0.25% Trypsin–EDTA. The resulting cell suspensions were stained with 1:20 mouse Alexa Fluor 488 anti-TNAP (BD Biosciences, #561495; RRID: AB_10897143) and 1:100 rat PE/Cyanine7 anti-PDPN (BioLegend, San Diego, CA, USA, # 337013; RRID: AB_2563367) antibodies for 15 min. Human PGCLCs were sorted with a SH800 Cell Sorter (SONY, Tokyo, Japan) directly into RNA Lysis Buffer (Zymo Research, Irvine, CA, USA, #R1060-1-100). Cell sorting analyses were performed with the provided Cell Sorter Software (SONY).

### RNA-sequencing libraries preparation

Total RNA from 1000 to 15 000 pelleted hiPSCs or sorted hPGCLCs was extracted using the Quick-RNA Microprep Kit (Zymo Research, #R2060) following the manufacturer’s instructions. Briefly, cells were resuspended and lysed in 150 µl of RNA Lysis Buffer, and an equal volume of ethanol was added. Total RNA was then purified using extraction columns. The procedure included a DNase I treatment to remove residual DNA and specific buffer washes to ensure RNA purity. Finally, RNA was eluted in 12 µl of DNase/RNase-free water.

DNA libraries for next-generation sequencing were constructed from all samples using the Smart-seq2 protocol with 1 ng of total RNA for each sample (two replicates for each condition per patient). The enzymatic fragmentation and tagmentation of DNA were performed using the Nextera XT kit (Illumina Inc., San Diego, CA, USA, #FC-131-1024) along with IDT^®^ for Illumina^®^ DNA Unique Dual Index barcodes. The final amplified libraries were purified with AMPure XP beads at a ratio of 1:1 (sample versus beads). The final cDNA library was quantified using a Qubit 1X HS DNA assay kit (Thermo Fisher Scientific, #Q33230), and the quality of the libraries was analyzed using a 2100 Bioanalyzer system (Agilent, Santa Clara, CA, USA) with a high-sensitivity DNA chip (Agilent). An equimolar concentration of 20 ng from each library was pooled and sequenced on Illumina NovaSeq X Plus sequencing platform (Novogene, Planegg, Germany) with 150 bp paired-end read setup.

While well established and broadly regarded as sufficient for validating hPGCLC identity (Irie *et al.*, 2015; Sasaki *et al.*, 2015; Alves-Lopes *et al.*, 2023), we recognize that having fewer than three replicates per condition can represent a limitation of our transcriptomic analysis.

### Bioinformatics analysis

Quality control (QC) and initial data processing were performed in Partek flow software (version 12.7.0, Illumina). Raw fastq files were processed using the standard Partek flow pipeline for bulk transcriptomic data. Initially, a pre-alignment QC report was generated to assess overall read quality scores, base composition, sequence length, and overrepresented sequences. Next, low-quality bases and adapters were trimmed (CUTADAPT_4_2) from the 3′ end of each read where the Phred quality score was less than 20. Trimmed reads were aligned to the Hg38 human reference genome using the STAR aligner tool (STAR 2.7.8a), with a maximum mismatch setting of 1, and the remaining parameters set as default. Post-alignment QC was performed on the aligned reads to assess percentage mapped reads, coverage, and read distribution. Aligned reads were quantified to the hg38_ensembl_release113_v2 annotation model using the Partek Expectation–Maximization algorithm. Gene and transcript counts were filtered for noise reduction in Partek flow, excluding features where value <=1.0 in at least 80% of the samples. Filtered counts and sample metadata files were exported from Partek flow for downstream analyses in R (R version 4.4.2; Stockholm, Sweden).

Further sample QC and visualization were performed in R (counts per biotype, library sizes). Differential gene expression analysis was performed using the DESeq2 (v1.46.0) package in R. Raw count data were filtered to remove low-expressed genes, retaining only those with at least 10 counts in three or more samples. The DESeq2 dataset was constructed with design formula ∼ Protocol + Patient, and gene-wise dispersion estimates were computed using a negative binomial model. Normalized counts obtained via DESeq2’s variance stabilizing transformation (VST, blind = TRUE) were used for downstream visualization, including principal component analysis (PCA), and for heatmaps, where per-gene centering was performed by subtracting the mean expression across samples.

### Comparative analysis with published datasets

To facilitate comparative analysis with published *in vitro* and *in vivo* samples, raw data from (Alves-Lopes *et al.*, 2023) were retrieved from GEO (GSE203156) and processed using the same QC cutoffs and normalization parameters as used for our dataset. To account for technical variation between the two datasets, batch effect correction was performed on the integrated expression matrix using the remove BatchEffect function of the limma package in R. The batch corrected data were then used for dimensionality reduction. PCA was performed on the corrected counts to investigate biological clustering of samples across both datasets. Heat maps were generated using the same batch-corrected values.

## Results

### Establishment of primary testicular somatic cell cultures from cryopreserved tissue samples

Cryopreserved prepubertal testicular tissue fragments were carefully thawed and dissociated into single-cell suspensions using a combined enzymatic and mechanical approach. The resulting viable cells enabled the establishment of primary testicular somatic cell cultures from two prepubertal patient samples (Patients 1 and 2). These cultures displayed a myoid-like morphology and maintained stable proliferation across 7–8 passages (Fig. 1B, left panel). Early-passage primary testicular somatic cells from both patients were cryopreserved for downstream experiments and future analysis.

### Non-genome-integrating and feeder-free reprogramming of primary testicular somatic cells into hiPSCs

Early-passage primary testicular somatic cells were thawed and passaged once before being reprogrammed into hiPSCs using the non-genome-integrating StemMACS™ iPSC mRNA Reprogramming Kit. By the end of the protocol, at day 14, several emerging hiPSC colonies were visible in both reprogramming cultures (Fig. 1B, middle panel). Eight colonies per patient were manually picked to ensure biological redundancy and expanded as individual lines in primed pluripotency conditions (E8 medium) on laminin-521-coated plates without feeder cells. One representative hiPSC clone, NFKI001 for Patient 1 and NFKI002 for Patient 2, was selected for downstream characterization and analysis, based on optimal morphology and proliferation kinetics.

**Figure 2.**
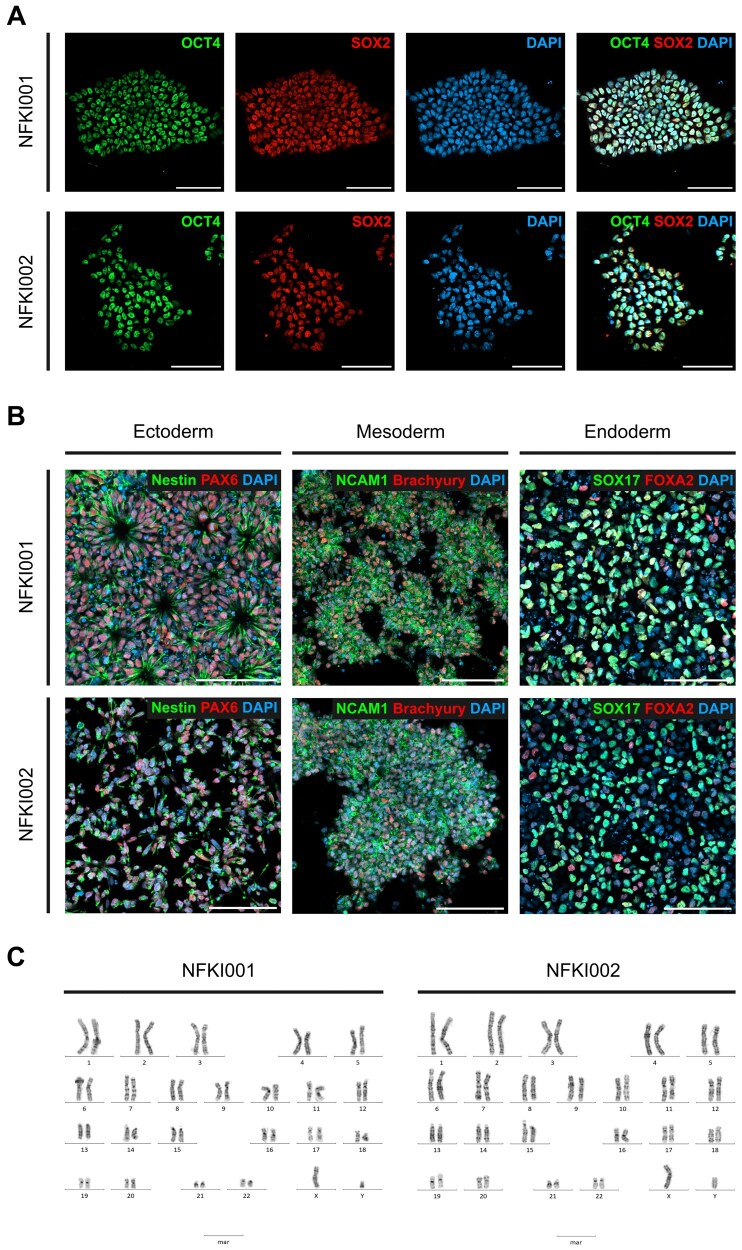
**Validation of hiPSC pluripotency, germ layer differentiation potential, and genomic stability**. (**A**) Immunofluorescence of pluripotency markers OCT4 and SOX2 on the generated NFKI001 and NFKI002 human induced pluripotent stem cell (hiPSC) lines. Nuclei were counterstained with DAPI. Scale bar, 100 μm. (**B**) Immunofluorescence of ectoderm markers Nestin and PAX6, mesoderm markers NCAM1 and Brachyury, and endoderm markers SOX17 and FOXA2 following directed differentiation to each germ layer. Nuclei were counterstained with DAPI. Scale bar, 100 μm. (**C**) Normal male karyograms from the generated hiPSC lines.

Both NFKI001 and NFKI002 lines exhibited typical hiPSC morphology, including a high nucleus-to-cytoplasm ratio and tightly packed colonies with smooth, well-defined edges (Fig. 1B, right panel). The validation of their pluripotency state was verified by the expression of the human pluripotency markers OCT4 and SOX2 (Fig. 2A). Furthermore, their pluripotent potential was confirmed by the trilineage differentiation assay using the STEMdiff™ Trilineage Differentiation Kit. After germ layer-specific differentiation, both lines expressed the ectoderm markers Nestin and PAX6, mesoderm markers Brachyury and NCAM1, and endoderm markers SOX17 and FOXA2 (Fig. 2B; Supplementary Fig. S1). Additionally, a normal 46, XY male karyotype was confirmed at p15 and p23 for NFKI001 and NFKI002, respectively (Fig. 2C).

### Patient-specific hiPSCs are competent for hPGCLC specification

**Figure 3.**
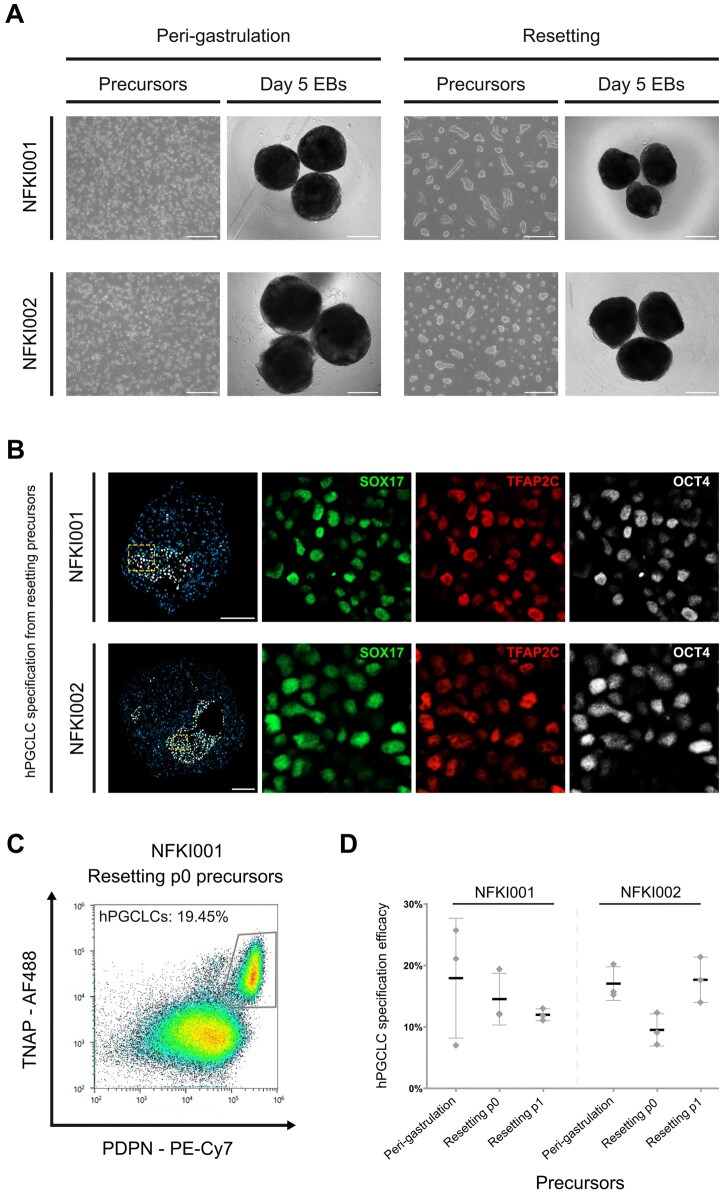
**Validation of hiPSC competence for hPGCLC specification**. (**A**) Phase-contrast images of the peri-gastrulation and resetting precursor cultures from both NFKI001 and NFKI002 human induced pluripotent stem cell (hiPSC) lines, along with their respective embryoid bodies (EBs) following 5 days of specification in human primordial germ cell-like cell (hPCGLC) induction media. Scale bar, 500 μm. (**B**) Immunofluorescence of hPGCLC markers SOX17, TFAP2C, and OCT4 from representative resetting precursor-derived EB sections for the NFKI001 and NFKI002 lines. The leftmost panel shows the merged channels with DAPI counterstaining (in blue) for the entire section, and the dashed box highlights the area enlarged in the right panels, displaying each individual channel. Scale bar, 100 μm. (**C**) Flow cytometry analysis plot showing the percentage of hPGCLCs co-expressing TNAP and PDPN in day 5 EBs generated from NFKI001 resetting passage 0 (p0) precursors. (**D**) Efficiency of hPGCLC specification (% of hPGCLCs within day 5 EBs) from peri-gastrulation, resetting p0 and resetting p1 precursors for both NFKI001 and NFKI002 lines. The horizontal black bars represent the mean percentage, and the vertical error bars represent the standard deviation, with n = 3 measurements for each condition. This experiment was designed to demonstrate the reproducibility of hPGCLC specification within each line and condition, rather than to compare efficiencies between lines or conditions.

The competence of NFKI001 and NFKI002 hiPSC lines to specify hPGCLCs was evaluated using two distinct protocols, differing in their starting precursor cell type: the PreME protocol, which starts with peri-gastrulation precursors (Kobayashi *et al.*, 2017), and the HENSM protocol, which starts with resetting precursors in a naïve-primed intermediary state of pluripotency (Bayerl *et al.*, 2021; Alves-Lopes *et al.*, 2023) (Fig 3A). Both precursor types from each hiPSC line were able to form embryoid bodies (EBs) during the 5-day differentiation protocol (Fig. 3A). We confirmed the presence of hPGCLCs in EB sections by immunodetecting the co-expression of the hPGC-specific markers SOX17, TFAP2C, and OCT4 (Fig. 3B; Supplementary Figs S2 and S3). Furthermore, hPGCLCs were quantified and isolated via fluorescence-activated cell sorting from all other somatic cell populations within the EBs based on the co-expression of TNAP and PDPN, both hPGC- and hPGCLC-specific cell surface markers (Fig. 3C; Supplementary Fig. S2). Upon triplicate hPGCLC inductions for each condition, NFKI001 generated 17.99% ± 9.79% (mean ± SD) TNAP+/PDPN+ hPGCLCs from peri-gastrulation, 14.56% ± 4.24% from resetting p0 precursors, and 12.00% ± 0.98% from resetting p1 precursors. NFKI002 produced 17.06% ± 2.76%, 9.50% ± 2.63%, and 17.69% ± 3.70% TNAP+/PDPN+ hPGCLCs under the same respective conditions (Fig. 3D).

### Patient-specific hiPSCs and hPGCLCs transcriptomic profile

To thoroughly characterize the transcriptomic profiles of the newly established NFKI001 and NFKI002 hiPSC lines, maintained under primed pluripotency conditions, and their derived hPGCLCs, originating from both resetting and peri-gastrulation precursors, we performed bulk RNA sequencing.

**Figure 4.**
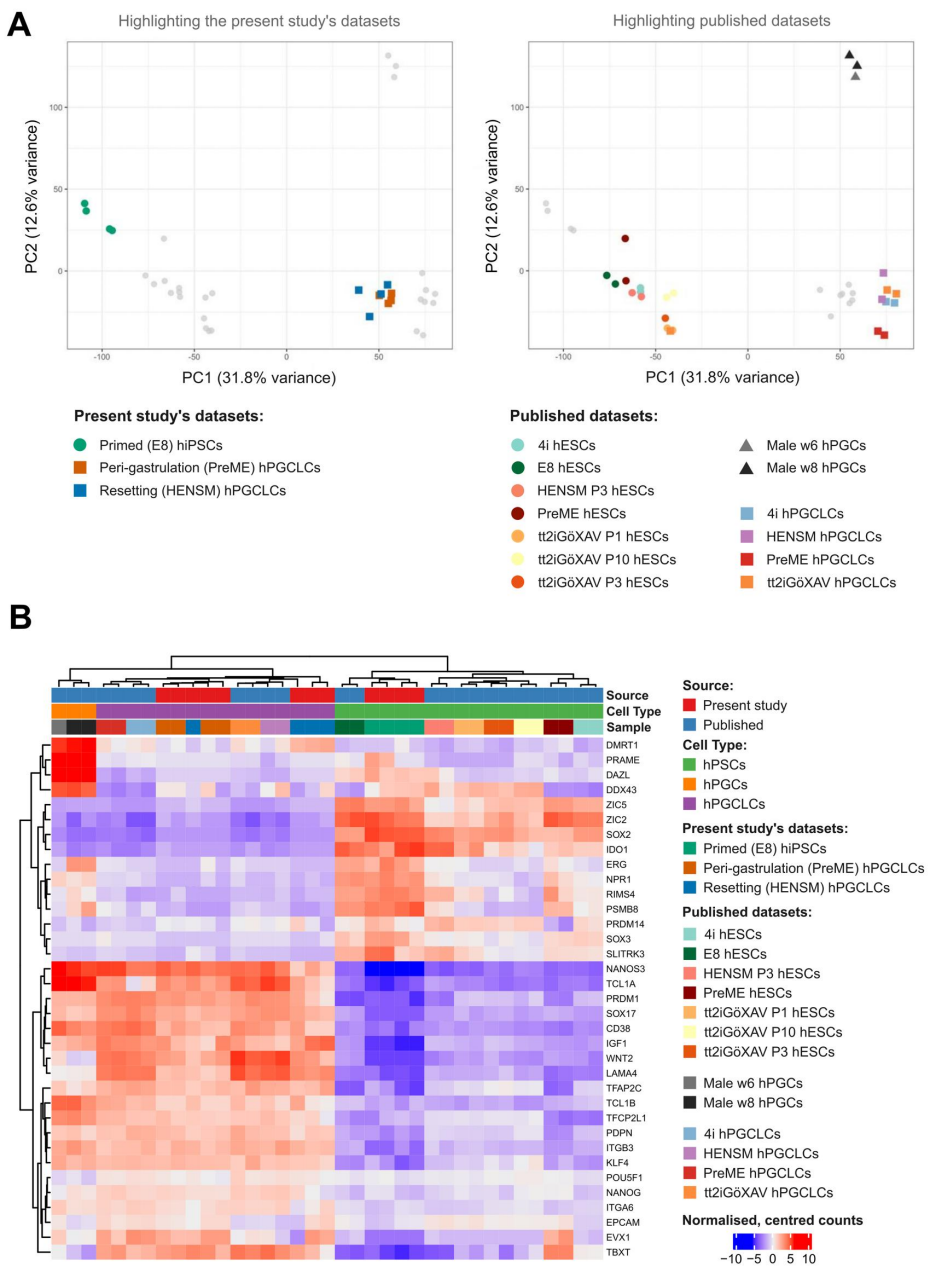
**Transcriptomic profile of hiPSC lines and derived hPGCLCs**. (**A**) Two-dimensional principal-component analysis (PCA; PC1 versus PC2) of primed human induced pluripotent stem cells (hiPSCs) and day 5 peri‑gastrulation and resetting human primordial germ cell-like cells (hPGCLCs) derived from NFKI001 and NFKI002 hiPSC lines (n = 2 replicates per condition). Samples from the present study were integrated with a previously published dataset (Alves-Lopes *et al*., 2023) comprising human embryonic stem cells (hESCs) cultured under primed (E8), peri‑gastrulation (4i and PreME), and resetting (HENSM and t2iGöXAV) conditions; hPGCLCs derived from peri‑gastrulation and resetting precursors; and *ex vivo* human male primordial germ cells (hPGCs) from developmental weeks 6 and 8 (n = 2 replicates per condition). For clarity, the PCA plot is split to highlight samples from the present and prior study. (**B**) Hierarchically clustered heatmap showing variance-stabilized transformed (VST) counts for selected pluripotency and hPGC marker genes across human pluripotent stem cells (hPSCs; either hiPSCs or hESCs), hPGCLCs, and hPGC samples from both present study and previously published datasets.

We first integrated the transcriptomic profiles of our samples with a previously published dataset (Alves-Lopes *et al.*, 2023) of hPSCs (primed, resetting, and peri-gastrulation states), hPGCLCs, and *ex vivo* hPGCs using principal component analysis (PCA) (Fig. 4A). We showed that NFKI001 and NFKI002 primed hiPSCs clustered together and aligned closely with the hPSC samples from the prior dataset. Crucially, they clearly separated from all hPGCLCs and hPGCs along principal component 1 (PC1), which accounts for 31.8% of the variance among our samples (Fig. 4A). Moreover, the hPGCLCs generated from NFKI001 and NFKI002 clustered together and in close proximity to the published hPGCLC samples, but remained distinctly separated from more mature *ex vivo* hPGCs along PC2, which represents 12.6% of the explained variance (Fig. 4A).

Subsequently, we evaluated the expression of genes associated with pluripotency and hPGC fate, revealing that NFKI001 and NFKI002 primed hiPSCs predominantly express pluripotency-associated genes (e.g. *SOX2*, *IDO1*, *ERG*, and *ZIC2*), whereas their derived hPGCLCs express genes linked to early (e.g. *NANOS3*, *SOX17*, *LAMA4*, and *TCL1A*), but not late (e.g. *DAZL*, *PRAME*, and *DDX43*) hPGC fate (Fig. 4B).

## Discussion

Infertility is recognized as one of the most significant long-term side effects of childhood cancer therapy (Duffin *et al.*, 2024). Early-stage clinical approaches using cryopreserved testicular tissue to restore fertility, including ectopic transplantation or SSC injection-based strategies, are currently under investigation (Kanbar *et al.*, 2022; Safrai *et al.*, 2025; Goossens *et al.*, 2026). However, the clinical success of these techniques relies on the presence of viable SSCs within the cryopreserved samples. When SSCs are compromised or absent, patient-specific hiPSC-based gametogenesis offers the most promising strategy for enabling biological parenthood.

In this study, we took the first steps toward addressing this challenge by successfully generating hiPSC lines from cryopreserved prepubertal testicular samples of childhood cancer patients with a severely depleted SSC pool. Most importantly, these patient-specific hiPSCs retained the capacity to initiate early embryonic gametogenesis, as demonstrated by their efficient differentiation into hPGCLCs using two distinct specification protocols. To the best of our knowledge, it represents the first description of cryopreserved prepubertal testis-derived hiPSC lines generated using a clinically compatible, non-genome-integrating reprogramming method, with subsequent differentiation into hPGCLCs.

Although the generation of testicular-derived hiPSCs has been previously explored, most studies have utilized viral-based reprogramming methods and non-GMP-compliant cultures. Kobayashi *et al.* (2011) generated hiPSC lines from freshly dissociated testicular cell suspensions obtained from two infertile adult patients, using a lentiviral delivery system on mouse supporting feeder cells. Subsequently, also relying on a viral protocol and feeder cells, Shimizu *et al.* (2016) reported the derivation of a hiPSC line from fresh testicular fibroblasts of a post-pubertal patient with Klinefelter syndrome, demonstrating its capacity for somatic differentiation into cardiomyocyte-like cells. More recently, two studies noted the feasibility of generating hiPSC lines from prepubertal testicular tissue using viral-based reprogramming, starting from cryopreserved testicular tissue of a patient with sickle cell disease and fresh tissue from a Klinefelter syndrome patient (Harancher *et al.*, 2023; Martin-Inaraja *et al.*, 2025). To date, only one study has reported hPGCLC specification from a patient-derived testicular hiPSC line, albeit from a post-pubertal individual with obstructive azoospermia. According to the authors, these hiPSCs generated with an episomal vector-based system yielded 15–20% EpCAM^+^/INTEGRINα6^+^ hPGCLCs via a peri-gastrulation precursor protocol (Wang *et al.*, 2019). In this context, our work further extends the efforts to obtain patient-derived germ cells by means of a recently developed specification protocol based on resetting precursors. This approach was shown to generate hPGCLCs bearing a developmentally enhanced potential compared to hPGCLCs generated from peri-gastrulation precursors (Alves-Lopes *et al.*, 2023).

The specification of validated hPGCLCs represented a significant milestone in the path to obtaining patient-specific hiPSC-derived gametes (Macedo and Alves-Lopes, 2026). Yet, hPGCLCs do not represent a cellular state with direct clinical applicability, further needing to recapitulate the remaining embryonic and foetal portions of gametogenesis, as well as the early postnatal development into the SSC state (Ramakrishna *et al.*, 2025). Current established maturation protocols have advanced hPGCLCs up to late embryonic mitotic prospermatogonia, although through xeno co-cultures with embryonic mouse testicular cells or relying on mouse feeder cells and xeno-conditioned medium (Hwang *et al.*, 2020; Murase *et al.*, 2024). Moreover, a recent study reported the presence of undifferentiated spermatogonia following transplantation of aggregates of hPGCLCs and embryonic mouse testicular cells into immunodeficient mice (Whelan *et al.*, 2024).

As the field moves towards a comprehensive understanding of the cellular and molecular mechanisms regulating male germline development and, in parallel, towards effectively obtaining *in vitro*-derived SSCs, there is a growing recognition that the emergence of hiPSC-derived gametes is an imminent reality (Le Goff *et al.*, 2024; de Bruin *et al.*, 2025; Macedo and Alves-Lopes, 2026). As such, it is imperative to develop protocols that meet the stringent safety standards required for clinical translation. While current hPGCLC maturation strategies that incorporate xenogeneic components represent the leading approach in modelling the human germline progression, their reliance on animal material raises some concerns regarding zoonotic transmission and immunogenicity (Subbiahanadar Chelladurai *et al.*, 2021). Accordingly, these maturation systems should be recreated in fully characterized, xeno-free platforms, such as bioengineered microfluidic and defined extracellular matrix-based systems, already under active investigation for other hiPSC-derived cell therapies (Lyra-Leite *et al.*, 2022).

Safety concerns extend beyond the maturation strategies and encompass the need for integrity checkpoints across all culture steps and cell states. To enable clinical applications, the entire pipeline, from hiPSC generation to SSC differentiation, should be optimized within a GMP-compliant framework that ensures sterility, identity, functionality, and genetic and epigenetic integrity by, for instance, whole-genome sequencing and genome-wide DNA methylation profiling (International Society for Stem Cell Research, 2025; Haase *et al.*, 2026). From our perspective, it will be essential to recapitulate the sequential germline development, with as many intermediary states as necessary (e.g. hPGCLCs, prospermatogonia, SSCs), and to rigorously validate their genetic, epigenetic, and transcriptomic profile against the corresponding *in vivo* counterparts (Macedo and Alves-Lopes, 2026).

Within the context of our approach, the genetic and epigenetic integrity, as well as the accessibility of the starting biological material, represent important considerations. In this study, we generated hiPSCs from cryopreserved prepubertal testicular samples obtained after exposure to cancer treatment, thereby demonstrating that our strategy is effective even when applied to particularly challenging starting material. However, recommended clinical practice involves cryopreserving prepubertal testicular tissue before cancer therapy or after minimal alkylating agent exposure to provide material with reduced exposure for downstream applications (Mitchell *et al.*, 2025). While hiPSCs could theoretically be generated from adult somatic cells on an as-needed basis later in life, for instance, from skin biopsies, these cryopreserved prepubertal tissues hold a lower age-related somatic mutation burden, thus constituting a preferential source of autologous cells for *in vitro* SSC generation (Vijg *et al.*, 2023). Moreover, utilizing a small fraction of this already-banked material eliminates the need for additional invasive procedures, highlighting the extensive value of existing biobanks of cryopreserved prepubertal testicular tissue for hiPSC-based fertility treatment approaches.

For cancer patients who did not undergo testicular biopsy during childhood, hiPSCs can instead be generated later in adulthood from alternative somatic tissues, such as skin or blood, obtained through less invasive procedures. However, waiting until adulthood to harvest these cells introduces the compounded risks of age-related mutations and prior chemotherapy exposure, which may hinder the genetic and epigenetic stability and long-term developmental fidelity of hiPSCs (Mitchell *et al.*, 2025). Therefore, we propose that a routine skin punch biopsy, performed prior to cancer treatment, should be established as a clinical recommendation for all prepubertal cancer patients, ensuring a young, low-mutation cell source for future hiPSC-based strategies. Importantly, regardless of the somatic source, age, and treatment state of the starting material, hiPSCs intended for downstream germline modelling or clinical translation must undergo comprehensive validation, enabling the identification and exclusion of aberrant cells. As a last resort alternative to restore fertility, screened-validated hiPSC-derived SSCs may even hold an advantage over endogenous SSCs that remain in the testis following cancer treatment, as these may harbour persistent undetectable abnormalities (Kaplanis *et al.*, 2022).

Beyond technical and safety challenges, the clinical application of hiPSC-derived SSCs also requires careful consideration of its bioethical implications. Key points include the establishment of an appropriate regulatory oversight to prevent potential misuse and addressing novel questions regarding legal parenthood (Cohen and Adashi, 2025). It also underscores risks of exacerbating reproductive inequities related to economic barriers and disability justice (National Academies of Sciences, Engineering, and Medicine, 2023). Proactive engagement among stakeholders, including researchers, clinicians, policymakers, and the general public, is therefore essential in guiding the clinical translation of hiPSC-derived SSCs.

In a broader context, hiPSCs generated from childhood cancer patients, including the ones generated from cryopreserved testicular samples, could serve as personalized models to investigate mechanisms of therapy‑induced cellular toxicity and drug resistance, identify early biomarkers of long‑term adverse effects, and screen for agents that prevent or reverse chemotherapy damage. Just as importantly, these hiPSC lines hold potential for applications beyond fertility restoration, serving as patient-specific platforms to model and address other cancer treatment-related complications. For instance, cardiotoxicity is a major life-threatening complication of cancer therapies that could be mitigated using hiPSC‑derived cardiomyocytes to model patient‑specific drug effects, screen cardioprotective compounds, and ultimately explore autologous cell‑replacement or regenerative strategies (Han *et al.*, 2017; Miyagawa *et al.*, 2022).

Taken together, our work establishes a foundation for patient-specific germline restoration by demonstrating that cryopreserved prepubertal testicular tissue with a severely depleted SSC pool can yield hiPSCs competent for hPGCLC specification. Moving forward, efforts should prioritize advancing hPGCLCs towards later germline stages, ensuring stringent genomic and epigenetic safeguards, and addressing ethical frameworks for clinical translation. By coupling existing biobank resources with stem cell technologies, we lay the groundwork for translating experimental advances into personalized, clinically viable solutions for fertility restoration and broader survivorship care.

## Supplementary Material

hoag054_Supplementary_Data

## Data Availability

The data underlying this article cannot be shared publicly to protect the privacy of individuals that participated in the study. The data will be shared on a reasonable request to the corresponding author.
