# Supplementary material for: Human iPSCs derived from cryopreserved testicular somatic cells enable germline regeneration in childhood cancer survivors
Source: Hum Reprod Open. 2026 Jun 3;2026(3):hoag054. doi: 10.1093/hropen/hoag054 (PMC13322296; doi:10.1093/hropen/hoag054)
Supplement: hoag054_Supplementary_Data [file hoag054_supplementary_data.pdf]

A

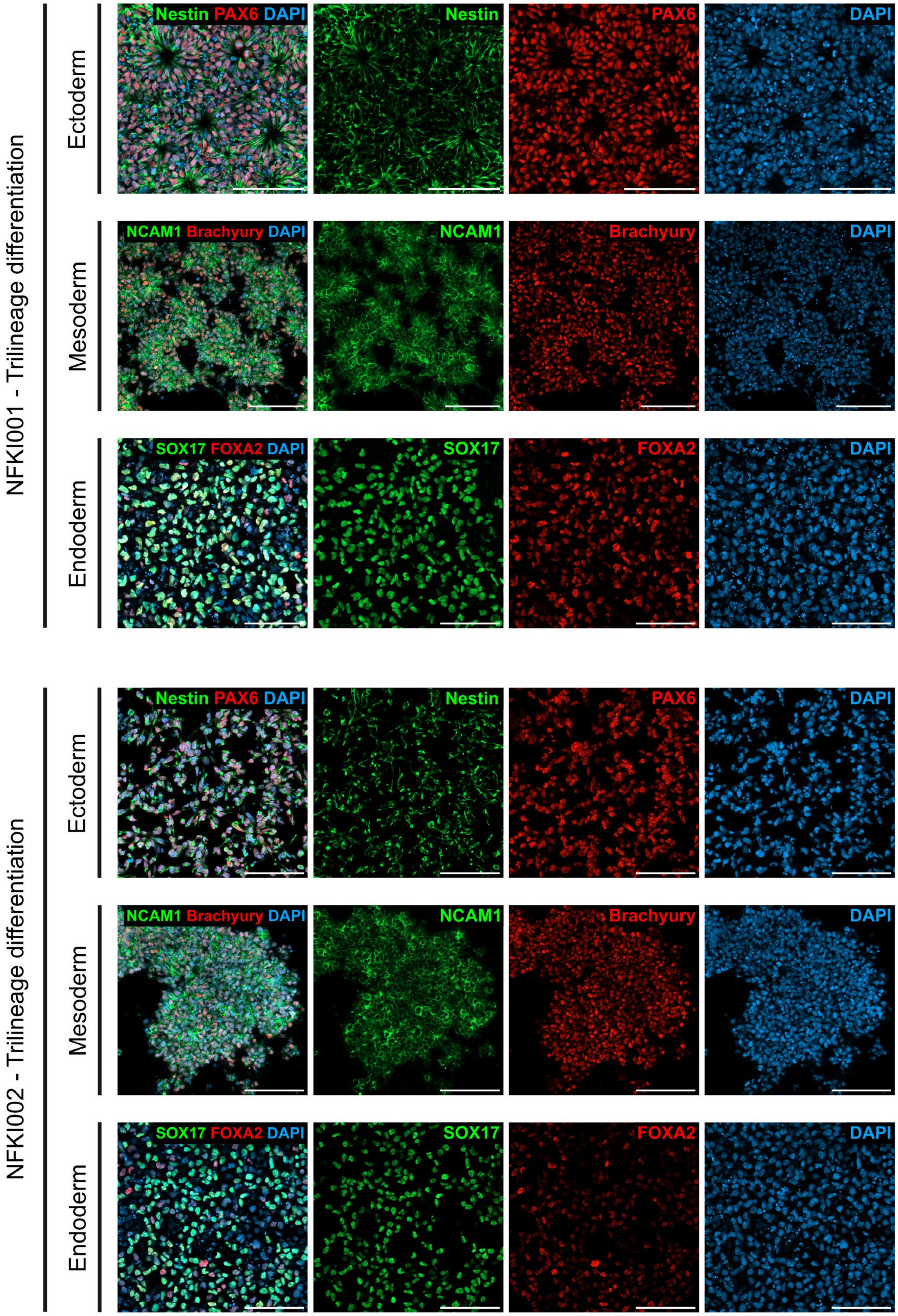
NFKI001 - Trilineage differentiation
Ectoderm
Nestin PAX6 DAPI
Nestin
PAX6
DAPI
Mesoderm
NCAM1 Brachyury DAPI
NCAM1
Brachyury
DAPI
Endoderm
SOX17 FOXA2 DAPI
SOX17
FOXA2
DAPI
NFKI002 - Trilineage differentiation
Ectoderm
Nestin PAX6 DAPI
Nestin
PAX6
DAPI
Mesoderm
NCAM1 Brachyury DAPI
NCAM1
Brachyury
DAPI
Endoderm
SOX17 FOXA2 DAPI
SOX17
FOXA2
DAPI

Supplementary Figure S1. Validation of hiPSC germ layer differentiation potential (channel split). (A) Immunofluorescence of ectoderm markers Nestin and PAX6, mesoderm markers NCAM1 and Brachyury, and endoderm markers SOX17 and FOXA2 following directed differentiation to each germ layer. Nuclei were counterstained with DAPI. Scale bar, 100 μm.

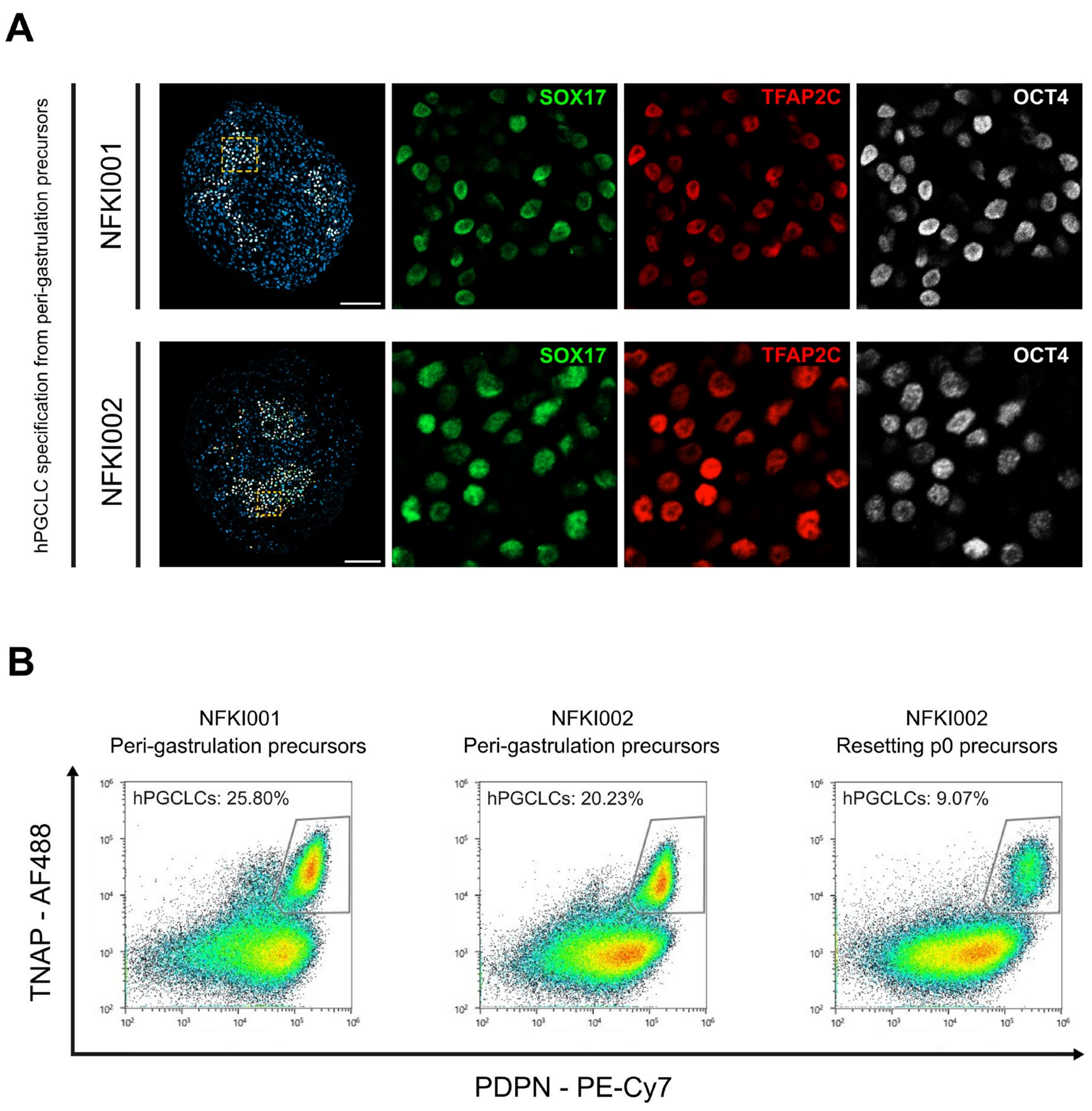

Supplementary Figure S2. Validation of hiPSC competence for hPGCLC specification (continuation). (A) Immunofluorescence of hPGCLC markers SOX17, TFAP2C and OCT4 from a representative peri-gastrulation precursor-derived embryoid-body (EB) section for NFKI001 and NFKI002 lines. The leftmost panel shows the merged channels with DAPI counterstaining (in blue) for the entire section, and the dashed box highlights the area enlarged in the right panels displaying each individual channel. Scale bar, 100 µm; **(B)** Flow cytometry analysis plot showing the percentage of hPGCLCs co-expressing TNAP and PDPN in day 5 EBs generated from NFKI001 peri-gastrulation precursors and NFKI002 peri-gastrulation, resetting passage 0 (p0) and resetting p1 precursors.

A

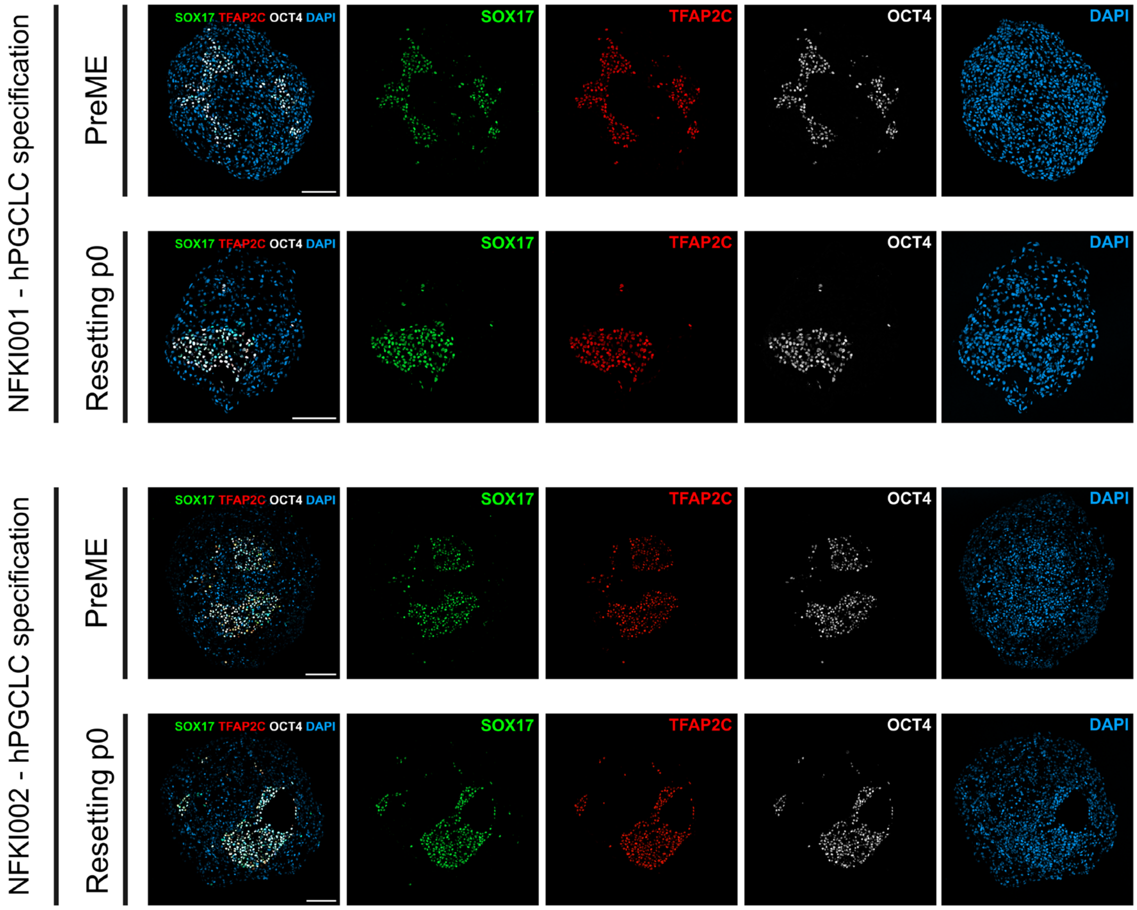

Supplementary Figure S3. Validation of hiPSC competence for hPGCLC specification (channel split). (A) Immunofluorescence of hPGCLC markers SOX17, TFAP2C and OCT4 from representative resetting precursor-derived EB sections for the NFKI001 and NFKI002 lines. Nuclei were counterstained with DAPI. Scale bar, 100 µm.
